# Doravirine Exposure and HIV-1 Suppression after Switching from an Efavirenz-Based Regimen to Doravirine-Lamivudine-Tenofovir Disoproxil Fumarate

**DOI:** 10.1128/AAC.01298-19

**Published:** 2019-11-21

**Authors:** Wayne Greaves, Hong Wan, Ka Lai Yee, Bhargava Kandala, Pavan Vaddady, Carey Hwang

**Affiliations:** a Global Clinical Development Infectious Diseases, Merck & Co., Inc., Kenilworth, New Jersey, USA; b Biostatistics and Research Decision Sciences, Merck & Co., Inc., Kenilworth, New Jersey, USA; c Pharmacokinetics, Pharmacodynamics, and Drug Metabolism, Merck & Co., Inc., Kenilworth, New Jersey, USA

**Keywords:** HIV-1, doravirine, efavirenz, efficacy, pharmacokinetics

## Abstract

Doravirine is a nonnucleoside reverse transcriptase inhibitor that has been approved for the treatment of HIV-1. In a phase 1 trial, doravirine exposure was transiently decreased when treatment was started immediately after the cessation of efavirenz treatment. In a *post hoc* subgroup analysis of participants who switched from an efavirenz-based regimen to doravirine-lamivudine-tenofovir disoproxil fumarate in the phase 3 DRIVE-SHIFT trial, doravirine plasma levels at week 4 were similar to noninduced levels, and HIV-1 suppression was maintained at weeks 24 and 48.

## Abstract (precis)

Doravirine is a nonnucleoside reverse transcriptase inhibitor that has been approved for the treatment of HIV-1. In a phase 1 trial, doravirine exposure was transiently decreased when treatment was started immediately after the cessation of efavirenz treatment.

## TEXT

Doravirine (DOR) is a novel nonnucleoside reverse transcriptase inhibitor (NNRTI) that has been approved for the treatment of HIV-1 at a dosage of 100 mg once daily. DOR (100 mg) is available as a single-entity tablet (1) and as a fixed-dose combination tablet with lamivudine (3TC) (300 mg) and tenofovir disoproxil fumarate (TDF) (300 mg) (2). The efficacy and safety of DOR in treatment-naive adults with HIV-1 were demonstrated in two phase 3 clinical trials, DRIVE-FORWARD (3) and DRIVE-AHEAD (4). More recently, maintenance of HIV-1 suppression was demonstrated in adults who switched from a stable antiretroviral regimen to DOR-3TC-TDF in another phase 3 clinical trial, DRIVE-SHIFT (5).

DOR may be an effective alternative for patients who do not tolerate efavirenz, a commonly used NNRTI that is included in many HIV-1 treatment regimens. The predominant route of elimination for DOR is oxidative metabolism mediated primarily by cytochrome P450 3A4 (CYP3A4) (6). Because efavirenz is a moderate inducer of CYP3A, a drug-drug interaction study was previously conducted in healthy adults to assess the pharmacokinetics of both drugs following a switch from efavirenz to DOR (7). In that study, plasma concentrations of DOR on day 1 and day 14 after a switch from efavirenz were lower than those determined in the absence of prior efavirenz treatment. The DOR trough concentration reached the *in vitro*-based target for inhibition of wild-type HIV-1 (78 nM) on day 2 after efavirenz cessation, while efavirenz was present at therapeutic concentrations (>1,000 ng/ml) until day 4. To understand the clinical relevance of this interaction, we conducted *post hoc* analyses of DOR plasma levels and the maintenance of viral suppression in participants who switched from an efavirenz-based regimen to DOR-3TC-TDF in the DRIVE-SHIFT clinical trial.

### Study design and participants.

The DRIVE-SHIFT study was an open-label, active-control, noninferiority trial in adults with HIV-1 who had experienced virological suppression for at least 6 months with two nucleoside reverse transcriptase inhibitors (NRTIs) plus a boosted protease inhibitor, boosted elvitegravir, or an NNRTI (5). The protocol was approved by the independent ethics committee for each study site, and all participants provided written informed consent before any study procedures were performed. Participants were randomly assigned, in a 2:1 ratio, to switch to once-daily DOR-3TC-TDF on day 1 (immediate switch group [ISG]) or to continue their current therapy and to switch to DOR-3TC-TDF at week 24 (delayed switch group [DSG]). Of the 670 participants who entered the trial, 114 (17%) were receiving an efavirenz-based regimen, and 556 (83%) were receiving two NRTIs with a boosted protease inhibitor, boosted elvitegravir, nevirapine, or rilpivirine. Baseline characteristics of these two groups were generally similar (Table 1).

**TABLE 1:** Baseline demographic and clinical characteristics

Characteristic	Previous efavirenz regimen (*n* = 114)	Other previous regimen (*n* = 556)^*a*^
Age (median [range]) (yr)	46 (24–71)	42 (21–71)
Male (no. [%])	98 (86.0)	468 (84.2)
Race/ethnicity (no. [%])		
White	78 (68.4)	434 (78.1)
Black or African American	18 (15.8)	72 (12.9)
Asian	7 (6.1)	18 (3.2)
Multiracial	6 (5.3)	29 (5.2)
Other race^*b*^	5 (4.4)	3 (0.5)
Hispanic or Latino	34 (29.8)	110 (19.8)
Region (no. [%])		
Asia/Pacific region	13 (11.4)	18 (3.2)
Europe	52 (45.6)	353 (63.5)
Latin America	18 (15.8)	55 (9.9)
North America	31 (27.2)	130 (23.4)
CD4^+^ T-cell count		
Median (range) (cells/mm^3^)	633 (184–1711)	626.5 (82–1928)
No. (%) with <200 cells/mm^3^	1 (0.9)	16 (2.9)
No. (%) with ≥200 cells/mm^3^	112 (98.2)	530 (95.3)
Duration of prior regimen		
Median (range) (mo)	65.1 (7.0–264.9)	46.9 (6.9–217.6)
No. (%) with duration of ≥12 mo	107 (93.9)	525 (94.4)
History of AIDS (no. [%])	20 (17.5)	95 (17.1)
Hepatitis B and/or C positive (no. [%])	3 (2.6)	20 (3.6)

a Other previous regimens included two NRTIs with nevirapine, rilpivirine, boosted elvitegravir, or a boosted protease inhibitor (atazanavir, darunavir, or lopinavir).

b Other race included American Indian and Alaska Native.

### Pharmacokinetics.

DOR plasma concentrations were measured for the 447 participants who switched to DOR-3TC-TDF on day 1; the previous antiretroviral regimen included efavirenz for 78 of those participants. Plasma samples for determination of DOR concentrations were collected before dosing at weeks 4, 24, and 48. DOR plasma concentrations were determined by Q^2^ Solutions (Morrisville, NC), using reverse-phase ultraperformance liquid chromatography with tandem mass spectrometric detection (lower limit of quantification, 1.00 ng/ml) (8).

**FIG 1:**
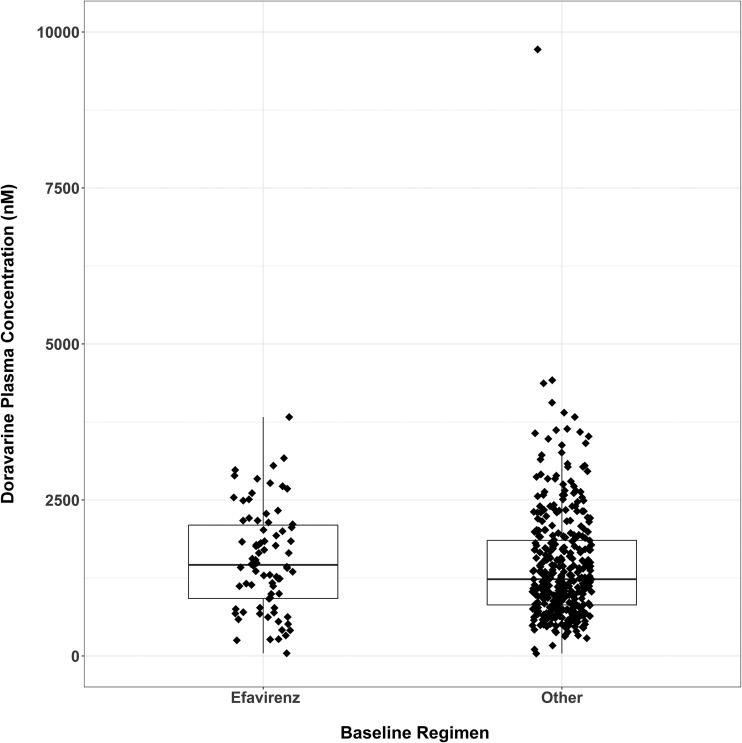
DOR plasma concentrations collected at study week 4 (before dosing) according to the baseline regimen (efavirenz versus other) in the ISG. The boxplot was overlaid with observed data points. Boxes denote 25th, 50th, and 75th percentiles, and the whiskers denote 1.5 times the interquartile range of distribution of predose samples.

**FIG 2:**
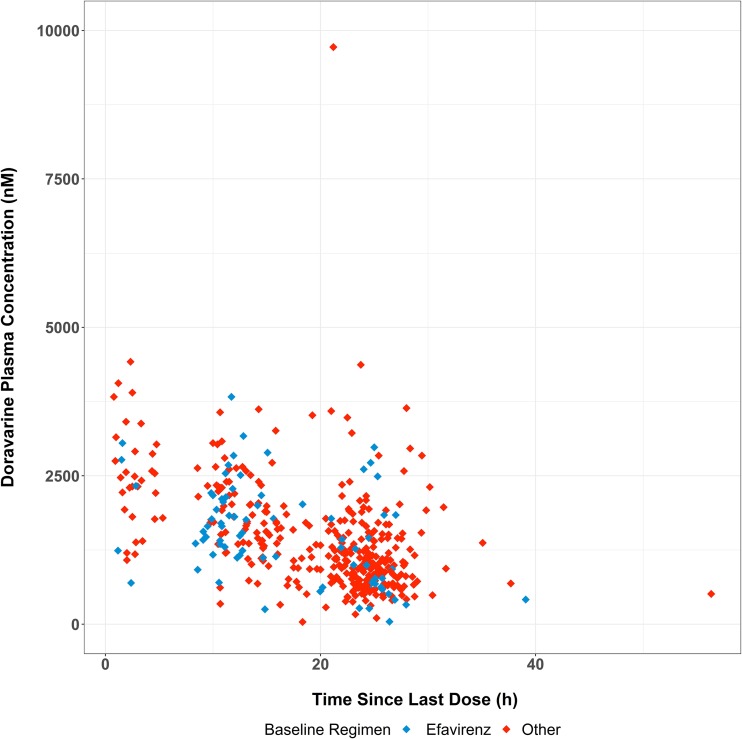
Individual plasma concentrations of DOR versus the actual time since the last dose at study week 4 in the ISG.

Plasma concentrations at week 4 were stratified according to the pretrial regimen (efavirenz based versus other) and summarized according to nominal sample time (Fig. 1). At week 4, predose plasma concentrations of DOR in participants who switched from an efavirenz-based regimen were consistent with those in participants who switched from another baseline regimen. Since the actual sampling times varied depending on when the participant arrived at the study site, DOR concentrations at week 4 were also plotted against the actual times since the last dose (Fig. 2). The concentration profiles were similar between the two groups, supporting the comparison based on nominal times.

### Clinical efficacy.

The primary efficacy endpoint in the DRIVE-SHIFT study was the proportion of participants with <50 copies/ml HIV-1 RNA at the primary time points of week 48 for the ISG and week 24 for the DSG, with the secondary time point of week 24 for both groups. The proportion of participants with ≥50 copies/ml HIV-1 RNA was a secondary endpoint and was also assessed at the primary and secondary time points. The efficacy analyses used the FDA Snapshot approach, which counts all missing data as failures regardless of the reason. The differences between treatment groups and the associated 95% confidence intervals (CIs) were calculated using the stratum-adjusted Mantel-Haenszel method.

The antiretroviral efficacy of DOR-3TC-TDF was similar for ISG participants who switched from an efavirenz-based regimen and those who switched from another baseline regimen (Table 2). At weeks 24 and 48, <50 copies/ml HIV-1 RNA was achieved in 97.4% and 93.6%, respectively, of the ISG participants who switched from an efavirenz-based regimen, compared with 93.0% and 90.2% of those who switched from another baseline regimen. Regarding the secondary endpoint, the proportions of participants with ≥50 copies/ml HIV-1 RNA at weeks 24 and 48 were 1.3% and 0.0%, respectively, of the ISG participants who switched from efavirenz, compared with 1.9% and 1.9% of those who switched from another baseline regimen. Only 1 ISG participant who switched from efavirenz had ≥50 copies/ml HIV-1 RNA at week 24, with a reported value of 51 copies/ml; the participant had <50 copies/ml HIV-1 RNA at all other time points in the study, including week 48. Only 1 participant with ≥50 copies/ml HIV-1 RNA had sufficient virus for resistance testing. That participant had switched from a non-efavirenz-based regimen and was discontinued from the study at week 36 due to lack of efficacy. No DOR resistance mutations were identified in that participant.

**TABLE 2:** Efficacy outcomes (FDA Snapshot approach)

Group^*a*^	No./total no. (% [95% CI])		Difference (% [95% CI])
	ISG	DSG	
<50 copies/ml HIV-1 RNA			
Week 24			
EFV	76/78 (97.4 [91.0 to 99.7])	36/36 (100 [90.3 to 100])	−2.6 (−8.0 to 2.9)
Non-EFV	343/369 (93.0 [89.8 to 95.3])	175/187 (93.6 [89.1 to 96.6])	−0.8 (−5.4 to 3.8)
ISG week 48 vs DSG week 24			
EFV	73/78 (93.6 [85.7 to 97.9])	36/36 (100 [90.3 to 100])	−6.4 (−13.3 to 0.4)
Non-EFV	333/369 (90.2 [86.7 to 93.1])	175/187 (93.6 [89.1 to 96.6])	−3.3 (−8.1 to 1.5)
≥50 copies/ml HIV-1 RNA^*b*^			
Week 24			
EFV	1/78 (1.3 [0.0 to 6.9])	0/36 (0.0 [0.0 to 9.7])	1.3 (−3.6 to 6.2)
Non-EFV	7/369 (1.9 [0.8 to 3.9])	4/187 (2.1 [0.6 to 5.4])	−0.1 (−3.0 to 2.7)
ISG week 48 vs DSG week 24			
EFV	0/78 (0.0 [0.0 to 4.6])	0/36 (0.0 [0.0 to 9.7])	0.0 (−4.2 to 4.2)
Non-EFV	7/369 (1.9 [0.8 to 3.9])	4/187 (2.1 [0.6 to 5.4])	−0.2 (−3.1 to 2.6)

a EFV, efavirenz.

b Included participants who changed any component of background therapy to a new drug class, background components that were not permitted according to the protocol, or any background drug in the regimen because of lack of efficacy (perceived or documented) before study week 24, participants who discontinued the study drug or the study before study week 48 because of lack or loss of efficacy, and participants with ≥50 copies/ml HIV-1 RNA in the time window.

These *post hoc* analyses of data from the phase 3 DRIVE-SHIFT trial showed that plasma levels of DOR after 4 weeks of treatment with DOR-3TC-TDF were not different for participants who switched from an efavirenz-based regimen and those who switched from a protease inhibitor, elvitegravir, or another NNRTI-based regimen. Because DOR plasma concentrations were not measured until 4 weeks after the initiation of therapy, our analyses did not address whether DOR exposure was reduced in the immediate period after the switch from efavirenz, as was observed in the phase 1 drug interaction study (6). Our analyses also showed that the antiretroviral efficacy of DOR at week 24 and week 48 was similar for participants who switched from an efavirenz-based regimen and those who switched from another baseline regimen. Thus, the antiretroviral efficacy of DOR was not adversely affected by prior treatment with efavirenz.

## References

[1] Merck Sharp & Dohme Corp. 2018 Pifeltro prescribing information. Merck Sharp & Dohme Corp, Whitehouse Station, NJ.

[2] Merck Sharp & Dohme Corp. 2018 Delstrigo prescribing information. Merck Sharp & Dohme Corp, Whitehouse Station, NJ.

[3] Molina J-M, Squires K, Sax PE, Cahn P, Lombaard J, DeJesus E, Lai M-T, Xu X, Rodgers A, Lupinacci L, Kumar S, Sklar P, Nguyen B-Y, Hanna GJ, Hwang C, Martins M, Cahn PE, Lopardo GD, Porteiro N, Bloch MT, Baker DA, Roth N, Moore RJ, Finlayson RJ, McMahon J, Rieger A, Zoufaly A, Hartl S, Zangerle R, Smaill F, Walmsley SL, Conway B, Rachlis A, Smith GHR, Perez C, Afani A, Campos Barker MIE, Chahin CE, Wolff Reyes M, Gerstoft J, Weis N, Laursen AL, Molina J-M, Yazdanpanah Y, Cotte L, Raffi F, Morlat P, Girard P-M, Katlama C, Rockstroh JK, Arasteh K, Esser S, Stoehr A, Stellbrink H-J, Stoll M, Schuermann D, Faetkenheuer G, Bogner J, Lutz T, Baumgarten A, Jaeger H, Gori A, Coltan G, Constandis F, Erscoiu SM, Prisacariu L-J, Rugina S, Streinu-Cercel A, Pokrovsky VV, Zakharova NV, Shuldyakov AA, Ryamova EP, Kulagin VV, Tsybakova OA, Orlova-Morozova E, Nagimova F, Voronin E, Shimonova TE, Kozyrev OA, Orrell C, Lombaard JJ, Botes ME, Portilla J, Gatell JM, Perez MJ, Arribas JR, Negredo E, Podzamczer D, Pulido F, Troya J, De los Santos I, Berenguer J, Williams IG, Johnson MA, Schembri G, Clarke A, Gompels M, Fox JM, Taylor SJ, Kegg S, Hagins DP, Osiyemi OO, Prelutsky DJ, Ramgopal MN, Dretler R, DeJesus E, Sloan L, Lewis ST, Clay PG, Bellos NC, Thompson MA, Montero J, McDonald CK, Creticos C, Shamblaw D, Terrelonge AE, Valdes M, Tashima KT, Robbins WJ, Felizarta FA, Elion RA, Slim J, Lalezari JP, Lalla-Reddy SN, Ruane PJ, Mills A, Cade JL, Campo RE, Dietz CA, Blick G, Mayer C, Rondon JC, Cook PP, Daar E, Kumar PN, Swindells S, Castro JG, Morales-Ramirez JO, Santiago L, Santana-Bagur JL 2018 Doravirine versus ritonavir-boosted darunavir in antiretroviral-naive adults with HIV-1 (DRIVE-FORWARD): 48-week results of a randomised, double-blind, phase 3, non-inferiority trial. Lancet HIV 5:e211–e220. doi:10.1016/S2352-3018(18)30021-3. 29592840

[4] Orkin C, Squires KE, Molina JM, Sax PE, Wong WW, Sussmann O, Kaplan R, Lupinacci L, Rodgers A, Xu X, Lin G, Kumar S, Sklar P, Nguyen BY, Hanna GJ, Hwang C, Martin EA 2019 Doravirine/lamivudine/tenofovir disoproxil fumarate is non-inferior to efavirenz/emtricitabine/tenofovir disoproxil fumarate in treatment-naive adults with human immunodeficiency virus-1 infection: week 48 results of the DRIVE-AHEAD Trial. Clin Infect Dis 68:535–544. doi:10.1093/cid/ciy540. 30184165 PMC6355823

[5] Johnson M, Kumar P, Molina JM, Rizzardini G, Cahn P, Bickel M, Mallolas J, Zhou Y, Morais C, Kumar S, Sklar P, Hanna GJ, Hwang C, Greaves W 2019 Switching to doravirine/lamivudine/tenofovir disoproxil fumarate (DOR/3TC/TDF) maintains HIV-1 virologic suppression through 48 weeks: results of the DRIVE-SHIFT Trial. J Acquir Immune Defic Syndr 81:463–472. doi:10.1097/QAI.0000000000002056. 30985556 PMC6905402

[6] Sanchez RI, Fillgrove KL, Yee KL, Liang Y, Lu B, Tatavarti A, Liu R, Anderson MS, Behm MO, Fan L, Li Y, Butterton JR, Iwamoto M, Khalilieh SG 2019 Characterisation of the absorption, distribution, metabolism, excretion and mass balance of doravirine, a non-nucleoside reverse transcriptase inhibitor in humans. Xenobiotica 49:422–432. doi:10.1080/00498254.2018.1451667. 29557716

[7] Yee KL, Sanchez RI, Auger P, Liu R, Fan L, Triantafyllou I, Lai MT, Di Spirito M, Iwamoto M, Khalilieh SG 2017 Evaluation of doravirine pharmacokinetics when switching from efavirenz to doravirine in healthy subjects. Antimicrob Agents Chemother 61:e01757-16. doi:10.1128/AAC.01757-16. PMC5278744 27872069

[8] Khalilieh S, Yee KL, Sanchez RI, Vaynshteyn K, Fan L, Searle S, Bouhajib M, Iwamoto M 2019 Evaluation of the pharmacokinetic interaction between doravirine and methadone. Clin Pharmacol Drug Dev doi:10.1002/cpdd.699. 31120195

